# Potential roles of PIWI-interacting RNAs in lung cancer

**DOI:** 10.3389/fonc.2022.944403

**Published:** 2022-10-17

**Authors:** Zheng Jian, Yichao Han, Hecheng Li

**Affiliations:** Department of Thoracic Surgery, Ruijin Hospital, Shanghai Jiao Tong University School of Medicine, Shanghai, China

**Keywords:** PIWI-interacting RNAs, biogenesis, lung cancer, tumorigenesis, mechanism

## Abstract

Lung cancer is a malignant tumor with high morbidity and mortality in the world today. Emerging evidence suggests that PIWI-interacting RNAs (piRNAs) are aberrantly expressed in various human cancers, including lung cancer. Despite of the poorly understood mechanism, piRNAs may work as carcinogenic roles or tumor suppressors by engaging in a variety of cancer-associated signaling pathways. Therefore, they might serve as potential therapeutic targets, diagnostic indicators, or prognostic indicators in lung cancer. This review will discuss the new findings of piRNAs, including their biosynthetic processes, mechanisms of gene suppression, and the significance of these piRNAs tested in lung cancer samples to determine their involvement in cancer progression.

## Introduction

Lung cancer is a common malignancy with high morbidity and mortality rates (1). More effective diagnostic and therapeutic approaches are still required. Numerous genetic and environmental factors may be involved in the formation of lung cancer (2). Internal factors such as genetic and epigenetic processes have emerged as critical contributors in lung cancer (2, 3). Non-coding RNA (ncRNA) is an epigenetic regulator influencing various cellular and molecular pathways (4). In recent studies, the biological role of ncRNA has been tentatively explored in lung-cancer-related studies (4, 5).

Only around 1% to 2% of the human genome sequence can be transcribed and translated into proteins, whereas the rest 98% or more are ncRNA (6). ncRNA is a type of RNA that does not include any protein-coding sequences and often contains small interfering RNA (siRNA), microRNA (miRNA), and PIWI-interacting RNA (piRNA). Among them, the piRNA is a novel family of non-coding short RNA (7, 8), and it is distinguished from miRNA and siRNA in downstream molecules: piRNA acts by binding to PIWI subfamily proteins, whereas miRNA and siRNA interact with AGO subfamily proteins. Nevertheless, siRNA, miRNA, and piRNA have shared function in regulating gene expression, i.e. silencing or initiating mRNA degradation (9, 10). Although piRNAs were initially recognized as a critical mechanism in germ cell maintenance (11, 12), an increasing number of researchers have shown that aberrant expressions of piRNA have been found in different kinds of tumors, and that the levels of PIWI expression are also strongly associated with tumor types (13–15). However, the specific role of piRNAs in cancer could be dual (either oncogenic or tumor-suppressive), and the manner of expression of piRNA in different types of tumors remains unknown and all require further investigation.

In this review, we will discuss the new findings of piRNAs in lung cancer, including their biosynthetic processes, mechanisms of gene suppression, as well as the significance of these piRNAs tested in lung cancer samples to determine their involvement in cancer progression.

## Overview of piRNA

### Transcription of piRNA

piRNA can be classified into unistrand and dual-strand clusters, both of which are transcribed by RNA polymerase II (16). Unistrand piRNA cluster products are dependent on conventional genic transcription units (particularly the conserved transcription factor A-MYB) and they will go through the canonical RNA processing in the nucleus, including 5’-capping, splicing, and polyadenylation (17, 18). Dual-strand piRNA clusters, in contrast are processed by non-canonical transcriptional pathways mediated by nuclear transcription factors such as Cutoff (Cuff), Deadlock (Del), and Rhino (Rhi) (19, 20).

**Figure 1:**
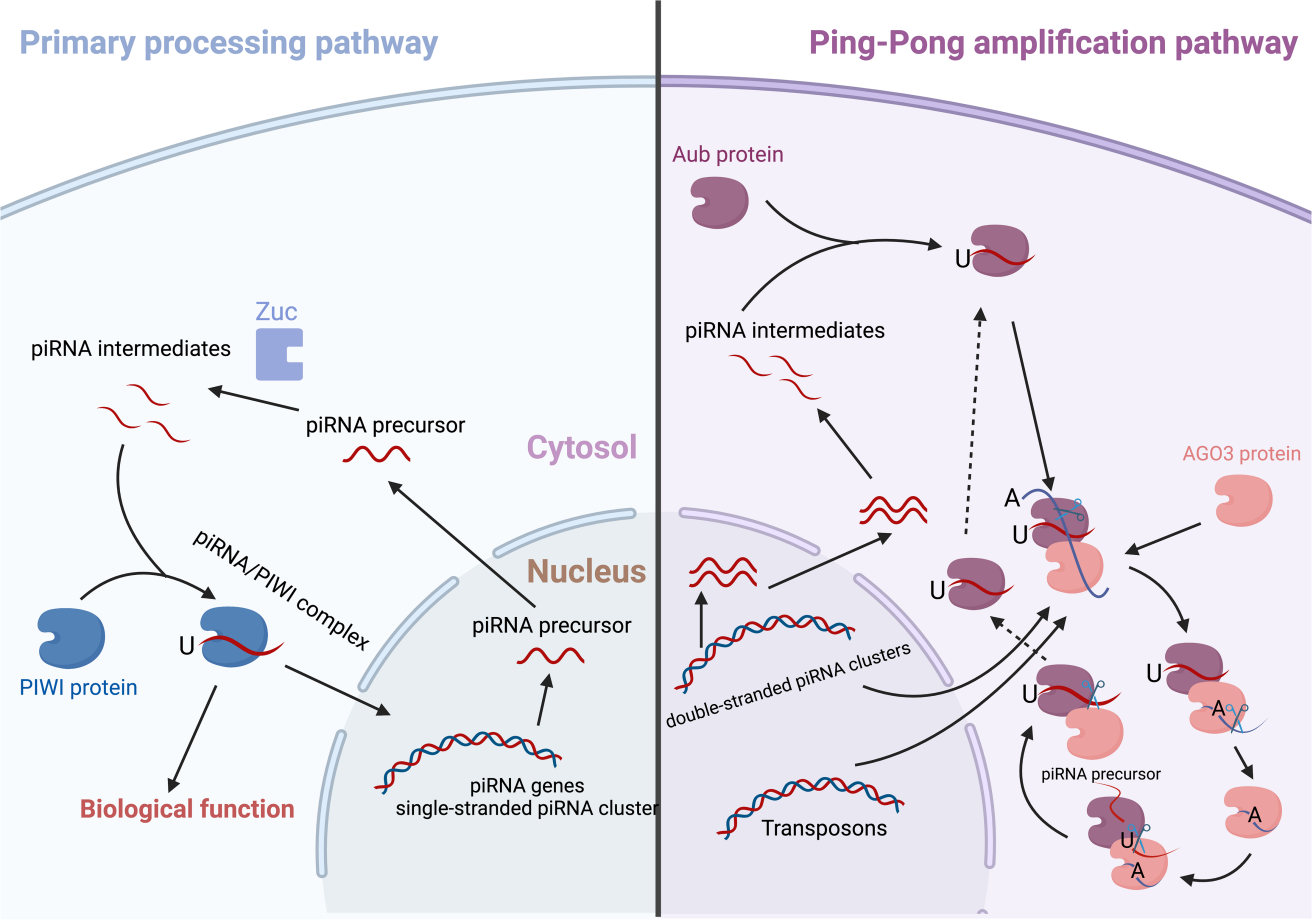
The biogenesis of piRNA/PIWI complex.

piRNA precursors are synthesized and then transported from the nucleus to the cytoplasm. After being cleaved and edited, the piRNAs intermediates combine with PIWI proteins to form the piRNAs-PIWI complex (21). Although the mechanisms for this biological process are complicated and have received limited attention, two major pathways have been identified, i.e. the primary processing pathway and the “Ping-Pong” amplification pathway (**Figure 1**).

### The primary processing pathway

The primary piRNAs are cleaved by an endoribonuclease enzyme called Zucchini (Zuc) after the RNA helicase Armitage unwinds their spirals (22). The 5’ uracil of sequences in piRNA intermediate then binds with a PIWI protein to become the intermediate piRNAs. Then, the 3’-end of the piRNA is trimmed to be its mature length by an Papi-dependent cleavage or Zuc riboendonuclease. In the end, the 2’-hydroxy group at the 3’-end is methylated by HEN1, resulting in a mature piRNA/PIWI complex (22, 23).

### The ping−pong amplification pathway

Once released from the nucleus, the precursor piRNAs will usually localize in the nuage, where they are involved in the biogenesis by a Ping-Pong cycle (24). The Ping-Pong cycle in *Drosophila* is dependent on Argonaute 3 (Ago3) and Aubergine (Aub) (25). A trigger piRNA will bind to Ago3 and then scan all transcripts that are released from the nucleus (26). After recognizing a target with a complementary sequence (antisense), the Ago3/piRNA complex attaches to and cleaves the precursor transcript, creating an antisense piRNA. The 5’-end of the cleaved transcript is subsequently converted into a responder piRNA (antisense) that recognizes Aub (26, 27). This complex may then proceed to edit the cluster transcripts (sense); the products from the edit process can restart the cycle (28). In the embryonic human ovaries, PIWIL2/HILI and PIWIL4/HIWI2 may establish a ping-pong cycle (28, 29).

### Biological functions of piRNA

In comparison with other noncoding RNAs, the functions of piRNAs in cell homeostasis and cancer progression are little known. In germ cells, piRNAs play a critical role in genome integrity maintenance, mRNA translation, and stability regulation, limiting DNA damage inflicted by transposable elements (TE) in genomic sequences (30–32).

piRNAs-mediated protection against transposon mobilization was initially found in fly germline cells. Similar function was also found in other species ranging from hydras to humans (33–37). Transposons, which are sometimes called “jumping genes” (38), can jeopardize gene stability by “copying and pasting” their own DNA into the host genome for self-replication, ultimately resulting in a cascade of undesired repercussions (38, 39). Transposons can alter transcriptions of target genes when their transcripts are inserted into the promoter or enhancer regions of target genes (40). Meanwhile, insertions into 5’ or 3’ UTRs may also have an effect on the post-transcriptional regulation of target genes (41). Sometimes, transposon insertions even can cause a DNA double-strand break, and failures to repair may result in recombination between transposon repetitions (42).

In the transcriptional control of gene expression, piRNAs can counteract the deleterious effects of TEs on the genome, preserving its integrity (30, 31). More specifically, DNA methylation in promoters of CpG islands can inhibit the initiation of DNA transcriptions, and piRNAs suppress the transcriptions of TEs by regulating the activities of DNA methyltransferases, such as DNMT1 or DNMT3A to maintain normal gametogenesis in germline cells (43). Additionally, modification of histone proteins is another underlying mechanism at the transcriptional level. The piRNA/PIWI complex can recruit histone methyltransferases to methylate the residues of histone lysine, such as H3K and H4K (44). However, the research to address the specifics of working mechanism remains limited, and plenty of difficult problems need to be resolved.

At the post-transcriptional level, piRNA/PIWI complex functions by binding to coding RNAs (mRNAs) or ncRNAs (45–47). Similar to silencing mechanism of miRNA, the piRNA/PIWI complex interacts with target RNA through efficient piRNA: RNA binding is formed by either a strict base pairing within 2~11 nt at the 5’ end of the piRNA (perfect pairing) or a less strict base pairing within 12~21 nt (imperfect pairing) (45). Then a piRNA induced silencing complex (piRISC) is developed which comprises RNAs and proteins after the base pairing is established. piRISC acts an important role in cleavage or deadenylation of target RNAs, which eventually leads to the decay of the target RNAs (46, 47).

### piRNA and cancer

Scientists have discovered critical shreds of evidence after the discovery of piRNA indicating the strong relationship between piRNA expression and numerous malignancies in recent years (48–50). For example, in one study, researchers have found that significant correlations between the expression of different kinds of piRNA and tumor metastasis, which was believed to further result in the dysregulation of signal cascades in cancer (51). Aberrant expression of PIWI has also been reported to be a contributing factor of cancer genesis. In many different types of tumors, such as breast cancer, colorectal cancer, liver cancer, the expression level of PIWI is higher than that in normal cells (52–54). PIWIL2, a member of the PIWI protein family, acts as a critical role in the process of piRNA biosynthesis, and it has been found absent in the majority of normal tissues from male adults, but it exists in a broad range of malignancies (55). Another typical example is PIWIL4, which was discovered in the testicular tissue and identified as a member of the PIWI protein subfamily. Normally, PIWIL4 is expressed in various human tissues, but it is considerably upregulated in cancer tissues (56). High expression of PIWIL4 is also reported to be associated with the initiation and progression of tumor (57).

### piRNA in lung cancer

Abnormal expression of several piRNAs has been discovered in lung cancer cells and tissues, demonstrating their potential correlation with the tumorigenesis and cancer progression (58–60). Li, et al. (58) reported that piR-651 was upregulated in lung cancer cell line assayed by real-time PCR (RT-PCR) and northern blot analysis by *in situ* hybridization. The increased expression of piR-651 in non-small cell lung cancer (NSCLC) was found to be associated with cancer progression in this study (58). Reeves, et al. identified over 500 piRNAs that were either up-regulated or down-regulated in lung cancer cell lines by piRNA microarray (61). Further analysis validated two upregulated piRNAs (piR-34871 and piR-52200) in lung cancer cells using RT-PCR and the aberrant expression of piR-34871 and piR-52200 were also confirmed in the lung cancer tissues and matched normal tissues (61). In some studies, expressions of piRNAs were found decreased in lung cancer. For example, Peng et al. showed that piR-55490 expressions was lower in the lung cancer specimens by RT-PCR analysis and found that there was a significant relevance between the overall survival rate and expression of piR-55490 in their research cohort (62).

Many piRNAs have been found in human lung cancer, but only a few of them, like piR-651, piR-55490, piR-211106, piR-34871, piR-52200, piR-46545, piR-35127, piR-26925, and piR-5444, have regulatory functions and molecular mechanisms, as detailed below (**Table 1**).

**Table 1:** Research reporting altered piRNA expression in lung cancer.

piRNA/ piR-Ls	Expression	Related Genes/Proteins	Functions	Sample types	Techniques	Clinical applications	References
piR-651	UP	CylinD1, CDK4, Caspace-3 Bcl-2, cleaved-PARP-1	Cell proliferation, apoptosis, migration and invasion	Tissues, Cell lines, Mice	piRNA microarray, RT-PCR	Diagnostic, Theraputic	(58, 63–65)
piR-55490	DOWM	mTOR	Cell proliferation and tumor progression	Tissues, Cell lines, Mice	RT-PCR	Theraputic	(62)
piR-211106	UP	PC	Drug resistance (apoptosis inhibition)	Tissues, Cell lines	RT-PCR	Theraputic	(66)
piR-46545	DOWN	RASSFIC, AMPK pathway	Cell proliferation	Tissues, Cell lines	piRNA microarray, RT-PCR	Theraputic	(61)
piR-35127	DOWN	RASSFIC,AMPK pathway	Cell proliferation	Tissues, Cell lines	piRNA microarray, RT-PCR	Theraputic	(61)
piR-34871	UP	RASSFIC, AMPK pathway	Cell proliferation	Tissues, Cell lines	piRNA microarray, RT-PCR	Theraputic	(61)
piR-52200	UP	RASSFIC,AMPK pathway	Cell proliferation	Tissues, Cell lines	piRNA microarray, RT-PCR	Theraputic	(61)
piR-26925	UP	–	Exosomal piRNAs in serum	Cell lines, Serum	piRNA microarray, RT-PCR	Diagnostic	(67)
piR-5444	UP	–	Exosomal piRNA in serum	Cell lines, Serum	piRNA microarray, RT-PCR	Diagnostic	(67)
piR-30074	–	–	Transformation of stem cells	Cell lines	–	Theraputic	(68)
piR-57125	DOWN	–	Distant metastasis	Tissues	RNA sequencing, RT-PCR	Prognostic	(69)
piR-L163	DOWN	ERM family, EBP-50, F-actin	Cell growth, and migration	Cell lines, Serum	small RNA sequencing, RT-PCR	Theraputic	(70)
piR-L138	UP	p60-MDM2	Drug-resistance (apoptosis inhibition)	Cell lines	RT-PCR	Theraputic	(71)

### piR-651

piR-651 is a type of piRNA first found in the pancreatic cancer, where it was demonstrated to regulate carcinogenesis through interaction with HIWI (63). Then Cheng et al. discovered that piR-651 was in high expression not only in pancreatic cancer cell lines, but also in liver, lung, colon, breast, and many other types of cancer cell lines (64). Further research confirmed that piR-651 was involved in the cell cycle and inhibiting piR-651 may arrest cancer cells in the G2/M phase (64). Another research discovered that overexpression of piR-651 significantly increased the viability and invasion of lung cancer cell lines (58). In this research, the proportion of cancer cells in G0/G1 phase was found lower in the presence of piR-651 than those in the absence of piR-651. Zhang et al. demonstrated that transfection of the piR651 inhibitor into the HCC827 and A549 cell lines inhibited cell proliferation by significantly increasing the rate of apoptosis, and that decreased the ability of cell migration compared to the control group (65). Furthermore, both *in vitro* and *in vivo* studies have shown that the levels of cyclin D1 and CDK4 were significantly connected with the levels of piR-651 expression (58, 65). In summary, these studies implied that piR651 was a valuable diagnostic biomarker and an effective therapeutic target in lung cancer.

### piR-55490

The expression of piR-55490 is downregulated in lung cancer, and several studies have shown that restoring piR-55490 can decrease lung cancer cell proliferation rates, whereas suppressing piR-55490 increases cell proliferation rates (62). In lung cancer cells, such as A549, piR-55490 acts as inhibiting the Akt/mTOR pathway, thereby suppressing cell growth. Further studies confirmed that piR-55490 was able to bind to the 3′-UTR of mTOR mRNA and inhibit its degradation by a mechanism similar to that of miRNAs (62). The presence of piRNA can contributed to the alteration of tumor cell phenotypes through regulating the decay of oncogenic mRNAs.

### piR-211106

The expression of piR-211106 is considerably decreased in NSLCLC tissues compared to normal tissues, indicating that piR-211106 may have an anti-oncogenic effect in lung cancer. A recent research discovered that piR-211106 inhibited pyruvate carboxylase (PC) at the mRNA and protein levels, as well as it directly interacting with the PC protein (66). The overexpression of piR-211106 results in a decrease in PC expression. However, the mechanism by which piR-211106 affects the expression of PC in NSCLC remains unknown.

It was also shown that piR-211106 was associated with treatment resistance in NSCLC cells (66). They discovered that cells overexpressing piR-211106 were more responsive to cisplatin, a first-line medication for advanced NSCLC. The pro-apoptotic effect of piR-211106 was believed to be one of underlying mechanism. Additionally, they found that piR-211106 functioned synergistically with cisplatin *in vitro* and *in vivo* (66). Due to the lack of efficacy of systemic chemotherapy in certain circumstances, effective local therapy is often advocated as a secondary treatment plan. This study demonstrated that directly injecting piR-211106 agomir into transplanted tumors in mice could significantly reduce tumor formation, suggesting that piR-211106 agomir might be a feasible treatment option for NSCLC.

### piR-46545, piR-35127, piR-34871 and piR-52200

The recognition of piRNAs in lung cancer cells overexpressing RASSF1C was shown by Reeves et al. (61). They observed that piR-52200 and piR-34871 expression levels were increased in half of the tested tumor tissues, and piR-46545 and piR-35127 expression levels were decreased. RASSF1C regulates the expression of these piRNAs as proven by microarray and real-time PCR. piR-52200 and piR-34871 silencing, as well as piR-46545 and piR-35127 overexpression, dramatically inhibited the proliferation of the H1299 and A549 cell lines (61). Thus, they concluded that these piRNAs might regulate lung cell transformation and carcinogenesis, and further studies confirmed that RASSF1C played an key role in piRNA target gene expression by blocking the AMPK pathway (61).

### piR-26925 and piR-5444

piR26925 and piR5444 expression levels were substantially higher in individuals with NSCLC than in healthy controls. Li et al. (67) reported that their values of area under curve(AUC)-ROC values were 0.751 and 0.713, respectively. To study their combined diagnostic application further, a multivariate logistic regression model using exosomal piR-26925 and piR-5444 was developed. In comparison to single piRNAs, the 2 piRNA panel had a stronger predictive power, with an AUC of 0.833 (95% confidence interval[CI]= 0.756~0.893, sensitivity = 87.1%, and specificity = 75.4%) (67). Additionally, they also discovered that multiple freeze–thaw cycles had no effect on the levels of piR-26925 and piR-5444 in serum exosomes from individuals with NSCLC (67). These findings support the use of serum exosomal piRNAs as possible diagnostic indicators of NSCLC.

### piR-L-163

piRNA-like small RNAs (piRNA-Ls) are emerging contributing factors in lung carcinogenesis and other pathological processes (70, 71)The expression of piRNA/piRNA-Ls in human lung bronchial epithelial cells and NSCLC cells was analyzed in a study (70). The data indicated that piR-L-163 might be involved in cell growth, proliferation, invasion, and migration by directly binding to and regulating phosphorylated ERM proteins, which were essentially required for the control of signal transduction pathways (71, 72).

### piR-L-138

piR-L-138 is another kind of piRNA-like small RNAs that plays a vital part in lung cancer formation and progression. *In vitro* and *in vivo* experiments showed that piR-L-138 expression was increased in response to chemoresistance to cisplatin (CDDP)-based treatment (71). piR-L-138 inhibition resulted in an increase in apoptosis in both CDDP-treated cell lines and xenograft animals models (71). MDM2 and its isoforms are involved in p53-independent apoptosis and chemoresistance (71, 73). In p53 mutant models, the interactions in the axis of piR-L-138/p60-MDM2 inhibited CDDP-induced apoptosis (73). Thus, elucidating piRNA-Ls functions and expanding our understanding of their capabilities may provide a feasible method to overcome chemotherapy resistance in patients with lung cancer.

## Conclusion

It is commonly accepted that the cancer cell is an unregulated somatic cell that has lost its normal regulatory mechanisms and reproduces uncontrollably (74–77). Given the resemblance between cancer stem cells and germ cells in terms of stemness and reproductive capabilities, the two cell types share several regulatory mechanisms (77). Although piRNAs were first identified as a class of non-coding short RNAs expressed specifically in the germline, accumulating data have demonstrated their abnormal expression in human malignancies (50, 51). Their corresponding biological functions in cancer cells can be varied and many, at the present stage, such as regulations in cancer cell proliferation, cell cycle, migration, invasion and drug-resistance (71, 75, 76).

While research on piRNAs in cancer fields represents a revolutionary chapter in the history of oncology, the majority of topics about piRNAs are currently poorly known in terms of their role in carcinogenesis and cancer progression. There are numerous critical obstacles related to piRNAs or piRNA/PIWI complex that we must overcome. For example, whether the biological synthesis and functions of piRNAs in cancer cells are comparable to those in germline cells; whether piRNAs are involved in the individual immunological mechanisms of cancer, since there is no evidence about the relation between piRNAs and immune checkpoint blockade drugs in cancer-therapy resistance; and more importantly, whether the identified dysregulated piRNAs in diverse types of cancer cells are genuine piRNAs because the ubiquitous background of short RNA sequencing data may mix with the whole databases, and some RNA fragments have been misidentified as piRNAs on occasion (78).

In conclusion, thorough knowledge of the carcinogenic/tumor suppressive role behind piRNAs is beneficial to open up new possibilities for the diagnosis and therapy of lung cancer. This current review may generate new ideas for future piRNA research, and we can be certain that more and more research will elucidate the particular biological processes through which piRNAs interact with cancer oncogenes and their potential roles as therapeutic targets, cancer-specific biomarkers or prognostic indicators in more details.

## Author contributions

ZJ and YH wrote the manuscript. All authors contributed to the article and approved the submitted version.

## Funding

This study was supported by National Natural Science Foundation of China (82072557, 81871882), National Key Research and Development Program of China (2021YFC2500900), Shanghai Municipal Education Commission- Gaofeng Clinical Medicine Grant (20172005) and program of Shanghai Academic Research Leader from Science and Technology Commission of Shanghai Municipality (20XD1402300).

## Conflict of interest

The authors declare that the research was conducted in the absence of any commercial or financial relationships that could be construed as a potential conflict of interest.

## Publisher’s note

All claims expressed in this article are solely those of the authors and do not necessarily represent those of their affiliated organizations, or those of the publisher, the editors and the reviewers. Any product that may be evaluated in this article, or claim that may be made by its manufacturer, is not guaranteed or endorsed by the publisher.

## References

[1] Siegel RL Miller KD Fuchs HE Jemal A . Cancer statistics, 2021. CA Cancer J Clin (2021) 71(1):7–33. doi: 10.3322/caac.21654 33433946

[2] Skoulidis F Heymach JV . Co-Occurring genomic alterations in non-small-cell lung cancer biology and therapy. Nat Rev Cancer (2019) 19(9):495–509. doi: 10.1038/s41568-019-0179-8 31406302 PMC7043073

[3] Fathizadeh H Asemi Z . Epigenetic roles of PIWI proteins and piRNAs in lung cancer. Cell Biosci (2019) 9:102. doi: 10.1186/s13578-019-0368-x 31890151 PMC6925842

[4] Huang J Peng J Guo L . Non-coding RNA: A new tool for the diagnosis, prognosis, and therapy of small cell lung cancer. J Thorac Oncol (2015) 10(1):28–37. doi: 10.1097/JTO.0000000000000394 25654726

[5] Valery PC Laversanne M Clark PJ Petrick JL McGlynn KA Bray F . Projections of primary liver cancer to 2030 in 30 countries worldwide. Hepatology (2018) 67(2):600–11. doi: 10.1002/hep.29498 PMC5832532 28859220

[6] Anastasiadou E Jacob LS Slack FJ . Non-coding RNA networks in cancer. Nat Rev Cancer (2018) 18(1):5–18. doi: 10.1038/nrc.2017.99 29170536 PMC6337726

[7] Liu Y Dou M Song X Dong Y Liu S Liu H . The emerging role of the piRNA/piwi complex in cancer. Mol Cancer (2019) 18(1):123. doi: 10.1186/s12943-019-1052-9 31399034 PMC6688334

[8] Tamtaji OR Behnam M Pourattar MA Hamblin MR Mahjoubin-Tehran M Mirzaei H . PIWI-interacting RNAs and PIWI proteins in glioma: molecular pathogenesis and role as biomarkers. Cell Commun Signal (2020) 18(1):168. doi: 10.1186/s12964-020-00657-z 33109195 PMC7590611

[9] Su JF Concilla A Zhang DZ Zhao F Shen FF Zhang H . PIWI-interacting RNAs: Mitochondria-based biogenesis and functions in cancer. Genes Dis (2020) 8(5):603–22. doi: 10.1016/j.gendis.2020.09.006 PMC8278532 34291132

[10] Riquelme I Pérez-Moreno P Letelier P Brebi P Roa JC . The emerging role of PIWI-interacting RNAs (piRNAs) in gastrointestinal cancers: An updated perspective. Cancers (Basel) (2021) 14(1):202. doi: 10.3390/cancers14010202 35008366 PMC8750603

[11] Brock M Mei Y . Protein functional effector sncRNAs (pfeRNAs) in lung cancer. Cancer Lett (2017) 403:138–43. doi: 10.1016/j.canlet.2017.06.013 28642173

[12] Enfield KS Martinez VD Marshall EA Stewart GL Kung SH Enterina JR . Deregulation of small non-coding RNAs at the DLK1-DIO3 imprinted locus predicts lung cancer patient outcome. Oncotarget (2016) 7:80957–66. doi: 10.18632/oncotarget.13133 PMC5348368 27829231

[13] Daugaard I Venø MT Yan Y Kjeldsen TE Lamy P Hager H . Small RNA sequencing reveals metastasis-related microRNAs in lung adenocarcinoma. Oncotarget (2017) 8(16):27047–61. doi: 10.18632/oncotarget.15968 PMC5432317 28460486

[14] Freedman JE Gerstein M Mick E Rozowsky J Levy D Kitchen R . Diverse human extracellular RNAs are widely detected in human plasma. Nat Commun (2016) 7:11106. doi: 10.1038/ncomms11106 27112789 PMC4853467

[15] Pekarsky Y Balatti V Palamarchuk A Rizzotto L Veneziano D Nigita G . Dysregulation of a family of short noncoding RNAs, tsRNAs, in human cancer. Proc Natl Acad Sci USA (2016) 113(18):5071–6. doi: 10.1073/pnas.1604266113 PMC4983805 27071132

[16] Goriaux C Desset S Renaud Y Vaury C Brasset E . Transcriptional properties and splicing of the flamenco piRNA cluster. EMBO Rep (2014) 15(4):411e418. doi: 10.1002/embr.201337898 24562610 PMC3989672

[17] Bolcun-Filas E Bannister LA Barash A Schimenti KJ Hartford SA Eppig JJ . A-MYB (MYBL1) transcription factor is a master regulator of male meiosis. Development (2011) 138(15):3319e3330. doi: 10.1242/dev.067645 21750041 PMC3133921

[18] Mohn F Sienski G Handler D Brennecke J . The rhinodeadlock-cutoff complex licenses noncanonical transcription of dual-strand piRNA clusters in drosophila. Cell (2014) 157(6):1364e1379. doi: 10.1016/j.cell.2014.04.031 24906153

[19] Li XZ Roy CK Dong X Bolcun-Filas E Wang J Han BW . An ancient transcription factor initiates the burst of piRNA production during early meiosis in mouse testes. Mol Cell (2013) 50(1):67e81. doi: 10.1016/j.molcel.2013.02.016 23523368 PMC3671569

[20] O¨zata DM Yu T Mou H Gainetdinov I Colpan C Cecchini K . Evolutionarily conserved pachytene piRNA loci are highly divergent among modern humans. Nat Ecol Evol (2020) 4(1):156-68. doi: 10.1038/s41559-019-1065-1 31900453 PMC6961462

[21] Williams Z Morozov P Mihailovic A Lin C Puvvula PK Juranek S . Discovery and characterization of piRNAs in the human fetal ovary. Cell Rep (2015) 13(4):854-863. doi: 10.1016/j.celrep.2015.09.030 26489470

[22] Zamore PD . Somatic piRNA biogenesis. EMBO J (2010) 29(19):3219–21. doi: 10.1038/emboj.2010.232 PMC2957218 20924396

[23] Bernstein BE Meissner A Lander ES . The mammalian epigenome. Cell (2007) 128(4):669–81. doi: 10.1016/j.cell.2007.01.033 17320505

[24] Grimson A Srivastava M Fahey B Woodcroft BJ Chiang HR King N . Early origins and evolution of microRNAs and piwi-interacting RNAs in animals. Nature (2008) 455(7217):1193–7. doi: 10.1038/nature07415 PMC3837422 18830242

[25] Wang W Han BW Tipping C Ge DT Zhang Z Weng Z . Slicing and binding by Ago3 or aub trigger piwi-bound piRNA production by distinct mechanisms. Mol Cell (2015) 59(5):819–30. doi: 10.1016/j.molcel.2015.08.007 PMC4560842 26340424

[26] Beyret E Liu N Lin H . piRNA biogenesis during adult spermatogenesis in mice is independent of the ping-pong mechanism. Cell Res (2012) 22(10):1429–39. doi: 10.1038/cr.2012.120 PMC3463270 22907665

[27] Gunawardane LS Saito K Nishida KM Miyoshi K Kawamura Y Nagami T . A slicer-mediated mechanism for repeat-associated siRNA 5′ end formation in drosophila. Science (2007) 315(5818):1587–90. doi: 10.1126/science.1140494 17322028

[28] Roovers EF Rosenkranz D Mahdipour M Han CT He N Chuva de Sousa Lopes SM . Piwi proteins and piRNAs in mammalian oocytes and early embryos. Cell Rep (2015) 10(12):2069-82. doi: 10.1016/j.celrep.2015.02.062 25818294

[29] Sasaki T Shiohama A Minoshima S Shimizu N . Identification of eight members of the argonaute family in the human genome. Genomics (2003) 82(3):323e330. doi: 10.1016/S0888-7543(03)00129-0 12906857

[30] Meseure D Alsibai KD . Part 1: The PIWI-piRNA p way is an immune-like surveillance process that controls genome integrity by silencing transposable elements. In: Logie C Knoch TA , editors. Chromatin and epigenetics. London, UK: IntechOpen (2018). p. 233–51. IntechOpen.

[31] Cheng Y Wang Q Jiang W . Emerging roles of piRNAs in cancer: Challenges and prospects. Aging (Albany NY). (2019) 11(21):9932-9946. doi: 10.18632/aging.102417 31727866 PMC6874451

[32] Moyano M Stefani G . piRNA involvement in genome stability and human cancer. J Hematol Oncol (2015) 8(1):38. doi: 10.1186/s13045-015-0133-5 25895683 PMC4412036

[33] Vagin VV Sigova A Li C Seitz H Gvozdev V Zamore PD . A distinct small RNA pathway silences selfish genetic elements in the germline. Science (2006) 313:320–4. doi: 10.1126/science.1129333 16809489

[34] Aravin AA Sachidanandam R Bourc’his D Schaefer C Pezic D Toth KF . A piRNA pathway primed by individual transposons is linked to de novo DNA methylation in mice. Mol Cell (2008) 31(6):785–99. doi: 10.1016/j.molcel.2008.09.003 PMC2730041 18922463

[35] Roovers EF Rosenkranz D Mahdipour M Han CT He N Chuva de Sousa Lopes SM . Piwi proteins and piRNAs in mammalian oocytes and early embryos. Nat Cell Biol (2021). 23(9):1013–22. doi: 10.1038/s41556-021-00750-6 25818294

[36] Praher D Zimmermann B Genikhovich G Columbus-Shenkar Y Modepalli V Aharoni R . Characterization of the piRNA pathway during development of the sea anemone nematostella vectensis. RNA Biol (2017) 14:1727–41. doi: 10.1080/15476286.2017.1349048 PMC5731801 28783426

[37] Gainetdinov I Skvortsova Y Kondratieva S Funikov S Azhikina T . Two modes of targeting transposable elements by piRNA pathway in human testis. RNA (2017). 23:1614–25. doi: 10.1261/rna.060939.117 PMC5648030 28842508

[38] Post C Clark JP Sytnikova YA Chirn GW Lau NC . The capacity of target silencing by drosophila PIWI and piRNAs. RNA (2014) 20(12):1977–86. doi: 10.1261/rna.046300.114 PMC4238361 25336588

[39] Ayarpadikannan S Kim HS . The impact of transposable elements in genome evolution and genetic instability and their implications in various diseases. Genomics Inform (2014) 12:98–104. doi: 10.5808/GI.2014.12.3.98 25317108 PMC4196381

[40] Li YZ Lu DY Tan WQ Wang JX Li PF . p53 initiates apoptosis by transcriptionally targeting the antiapoptotic protein ARC. Mol Cell Biol (2008) 28(2):564–74. doi: 10.1128/MCB.00738-07 PMC2223427 17998337

[41] Ernst C Odom D Kutter C . The emergence of piRNAs against transposon invasion to preserve mammalian genome integrity. Nat Commun (2017) 8(1):1411. doi: 10.1038/s41467-017-01049-7 29127279 PMC5681665

[42] Hedges DJ Deininger PL . Inviting instability: gransposable elements, double-strand breaks, and the maintenance of genome integrity. Mutat Res (2007) 616:46–59. doi: 10.1016/j.mrfmmm.2006.11.021 17157332 PMC1850990

[43] Kuramochi-Miyagawa S Watanabe T Gotoh K Totoki Y Toyoda A Ikawa M . DNA Methylation of retrotransposon genes is regulated by piwi family members MILI and MIWI2 in murine fetal testes. Genes Dev (2008) 22:908–17. doi: 10.1101/gad.1640708 PMC2279202 18381894

[44] Sugimoto K Kage H Aki N Sano A Kitagawa H Nagase T . The induction of H3K9 methylation by PIWIL4 at the p16 Ink4a locus. Biochem Biophys Res Commun 359:497–502. doi: 10.1016/j.bbrc.2007.05.136 17544373

[45] Goh WSS Falciatori I Tam OH Burgess R Meikar O Kotaja N . piRNA-directed cleavage of meiotic transcripts regulates spermatogenesis. Genes Dev (2015) 29:1032–44. doi: 10.1101/gad.260455.115 PMC4441051 25995188

[46] Rouget C Papin C Boureux A Meunier AC Franco B Robine N . Maternal mRNA deadenylation and decay by the piRNA pathway in the early drosophila embryo. Nature (2010) 467:1128–32. doi: 10.1038/nature09465 PMC4505748 20953170

[47] Watanabe T Lin H . Posttranscriptional regulation of gene expression by piwi proteins and piRNAs. Mol Cell (2014) 56:18–27. doi: 10.1016/j.molcel.2014.09.012 25280102 PMC4185416

[48] Chu H Xia L Qiu X Gu D Zhu L Jin J . Genetic variants in noncoding PIWI-interacting RNA and colorectal cancer risk. Cancer (2015) 121:2044–52. doi: 10.1002/cncr.29314 25740697

[49] Alexandrova E Lamberti J Saggese P Pecoraro G Memoli D Cappa VM . Small non-coding rna profiling identifies miR-181a-5p as a mediator of estrogen receptor beta-induced inhibition of cholesterol biosynthesis in triple-negative breast cancer. Cells (2020) 9(4):874. doi: 10.3390/cells9040874 PMC7226848 32260128

[50] Maleki DP Mansournia MA Mirhashemi SM . PIWI-interacting RNAs: new biomarkers for diagnosis and treatment of breast cancer. Cell Biosci (2020) 10:44. doi: 10.1186/s13578-020-00403-5 PMC7092456 32211149

[51] Busch J Ralla B Jung M Wotschofsky Z Trujillo-Arribas E Schwabe P . Piwi-interacting RNAs as novel prognostic markers in clear cell renal cell carcinomas[J]. J Exp Clin Cancer Res (2015) 34(1):61. doi: 10.1186/s13046-015-0180-3 26071182 PMC4467205

[52] Xu J Yang X Zhou Q Zhuang J Han S . Biological significance of piRNA in liver cancer: a review. Biomarkers (2020) 25(6):436–40. doi: 10.1080/1354750X.2020.1794041 32662667

[53] Weng W Liu N Toiyama Y Kusunoki M Nagasaka T Fujiwara T . Novel evidence for a PIWI-interacting RNA (piRNA) as an oncogenic mediator of disease progression, and a potential prognostic biomarker in colorectal:[J]. Mol Cancer (2018) 17(1):16. doi: 10.1186/s12943-018-0767-3 29382334 PMC5791351

[54] Kärkkäinen E Heikkinen S Tengström M Kosma VM Mannermaa A Hartikainen JM . The debatable presence of PIWI-interacting RNAs in invasive breast cancer. Cancer Med (2021) 10(11):3593–603. doi: 10.1002/cam4.3915 33960684 PMC8178507

[55] Erdogdu IH Yumrutas O Ozgur Cevik M Bozgeyik I Erdogdu M Inan HM . Differential expression of PIWIL2 in papillary thyroid cancers. Gene (2018) 649:8–13. doi: 10.1016/j.gene.2018.01.066 29369786

[56] Han YN Li Y Xia SQ Zhang YY Zheng JH Li W . PIWI proteins and PIWI-interacting RNA: Emerging roles in cancer. Cell Physiol Biochem (2017) 44(1):1–20. doi: 10.1159/000484541 29130960

[57] Ferreira HJ Heyn H Muro XGD Vidal A Larriba S Muñoz C . Epigenetic loss of the PIWI/piRNA machinery in human testicular tumorigenesis. Epigenet Off J DNA Methylation Soc (2014) 9(1):113–8. doi: 10.4161/epi.27237 PMC3928173 24247010

[58] Li D Luo Y Gao Y Yang Y Wang Y Xu Y . piR-651 promotes tumor formation in non-small cell lung carcinoma through the upregulation of cyclin D1 and CDK4. Int J Mol Med (2016) 38:927–36. doi: 10.3892/ijmm.2016.2671 27431575

[59] Liu J Zhang S Cheng B . Epigenetic roles of PIWIinteracting RNAs (piRNAs) in cancer metastasis (review). Oncol Rep (2018) 40:2423–34. doi: 10.3892/or.2018.6684 30226604

[60] Liang D Fang Z Dong M Liang C Xing C Zhao J . Effect of RNA interference-related HiWi gene expression on the proliferation and apoptosis of lung cancer stem cells. Oncol Lett (2012) 4:146–50. doi: 10.3892/ol.2012.677 PMC3398372 22807978

[61] Reeves ME Firek M Jliedi A Amaar YG . Identification and characterization of RASSF1C piRNA target genes in lung cancer cells. Oncotarget (2017) 8:34268–82. doi: 10.18632/oncotarget.15965 PMC5470966 28423657

[62] Peng L Song L Liu C Lv X Li X Jie J . piR-55490 inhibits the growth of lung carcinoma by suppressing mTOR signaling. Tumour Biol (2016) 37:2749–56. doi: 10.1007/s13277-015-4056-0 26408181

[63] Grochola LF Greither T Taubert H Moller P Knippschild U Udelnow A . The stem cell-associated hiwi gene in human adenocarcinoma of the pancreas: expression and risk of tumour-related death. Br J Cancer (2008) 99:1083–8. doi: 10.1038/sj.bjc.6604653 PMC2567072 18781170

[64] Cheng J Guo JM Xiao BX Miao Y Jiang Z Zhou H . piRNA, the new non-coding RNA, is aberrantly expressed in human cancer cells. Clinica Chimica Acta (2011) 412:1621–5. doi: 10.1016/j.cca.2011.05.015 21616063

[65] Zhang SJ Yao J Shen BZ Li GB Kong SS Bi DD . Role of piwi-interacting RNA-651 in the carcinogenesis of non-small cell lung cancer. Oncol Lett (2018) 15:940–6. doi: 10.3892/ol.2017.7406 PMC5772788 29399156

[66] Liu Y Dong Y He X Gong A Gao J Hao X . piR-hsa-211106 inhibits the progression of lung adenocarcinoma through pyruvate carboxylase and enhances chemotherapy sensitivity. Front Oncol (2021) 11:651915. doi: 10.3389/fonc.2021.651915 34249688 PMC8260943

[67] Li J Wang N Zhang F Jin S Dong Y Dong X . PIWI-interacting RNAs are aberrantly expressed and may serve as novel biomarkers for diagnosis of lung adenocarcinoma. Thorac Cancer (2021) 12(18):2468–77. doi: 10.1111/1759-7714.14094 PMC8447905 34346164

[68] Kozomara A Griffiths-Jones S . miRBase: annotating high confidence microRNAs using deep sequencing data. Nucleic Acids Res (2014) 42(Database issue):D68eD73. doi: 10.1093/nar/gkt1181 24275495 PMC3965103

[69] Daugaard I Veno MT Yan Y Kjeldsen TE Lamy P Hager H . Small RNA sequencing reveals metastasis-related microRNAs in lung adenocarcinoma. Oncotarget (2017) 8(16):27047-61. doi: 10.18632/oncotarget.15968 28460486 PMC5432317

[70] Mei Y Wang Y Kumari P Shetty AC Clark D Gable T . A piRNA-like small RNA interacts with and modulates p-ERM proteins in human somatic cells. Nat Commun (2015) 6:7316. doi: 10.1038/ncomms8316 26095918 PMC4557300

[71] Wang Y Gable T Ma MZ Clark D Zhao J Zhang Y . A piRNA-like small RNA induces chemoresistance to cisplatin-based therapy by inhibiting apoptosis in lung squamous cell carcinoma. Mol Ther Nucleic Acids (2017) 6:269–78. doi: 10.1016/j.omtn.2017.01.003 PMC5363509 28325293

[72] Morales FC Takahashi Y Kreimann EL Georgescu MM . Ezrin–radixinmoesin (ERM)-binding phosphoprotein 50 organizes ERM proteins at the apical membrane of polarized epithelia. Proc Natl Acad Sci U S A. (2004) 101:17705–10. doi: 10.1073/pnas.0407974101 PMC539771 15591354

[73] Zhang Z Li M Wang H Agrawal S Zhang R . Antisense therapy targeting MDM2 oncogene in prostate cancer: effects on proliferation, apoptosis, multiple gene expression, and chemotherapy. Proc Natl Acad Sci U S A. (2003) 100:11636–41. doi: 10.1073/pnas.1934692100 PMC208810 13130078

[74] Reya T Morrison SJ Clarke MF Weissman IL . Stem cells, cancer, and cancer stem cells. Nature (2001) 414:105e111. doi: 10.1038/35102167 11689955

[75] Li F Yuan P Rao M Jin CH Tang W Rong YF . piRNA-independent function of PIWIL1 as a co-activator for anaphase promoting complex/cyclosome to drive pancreatic cancer metastasis. Nat Cell Biol (2020) 22(4):425-38. doi: 10.1038/s41556-020-0486-z 32203416

[76] Niculescu VF . The reproductive life cycle of cancer: Hypotheses of cell of origin, TP53 drivers and stem cell conversions in the light of the atavistic cancer cell theory. Med Hypotheses (2019) 123:19–23. doi: 10.1016/j.mehy.2018.12.006 30696584

[77] Toh Tan B Lim JJ Chow EK-H . Epigenetics in cancer stem cells. Mol Cancer (2017) 16(1):e29. doi: 10.1186/s12943-017-0596-9 PMC5286794 28148257

[78] Tosar JP Rovira C Cayota A . Non-coding RNA fragments account for the majority of annotated piRNAs expressed in somatic non-gonadal tissues. Commun Biol (2018) 1:e2. doi: 10.1038/s42003-017-0001-7 PMC6052916 30271890

